# Severe Left Ventricular Outflow Obstruction by Native Anterior Mitral Leaflet After Bileaflet Preserving Mechanical Mitral Valve Replacement

**DOI:** 10.7759/cureus.80364

**Published:** 2025-03-10

**Authors:** Obieze Nwanna-Nzewunwa, Chinedu Okoli, Michael E Jessen, Suresh Keshavamurthy

**Affiliations:** 1 Cardiovascular and Thoracic Surgery, University of Texas Southwestern Medical Center, Hospitals and Health Care, Dallas, USA; 2 General Surgery, Maine Medical Center, Portland, USA; 3 Cardiovascular and Thoracic Surgery, University of Texas Southwestern Medical Center, Dallas, USA

**Keywords:** left ventricular outflow tract (lvot) obstruction, mitral valve replacement, pulmonary hypertension, systolic anterior movement (sam), tricuspid regurgitation

## Abstract

Left ventricular outflow tract obstruction (LVOTO) after mitral valve replacement (MVR) is a challenging complication. This case report describes a 70-year-old female who developed systolic anterior motion (SAM) secondary to an unresected anterior mitral leaflet several years following leaflet-preserving mitral valve replacement, with associated heart failure and tricuspid regurgitation. Surgical intervention included trans-aortic resection of the obstructing anterior mitral leaflet and chordae without re-replacing the mitral valve, followed by tricuspid valve bicuspidization. The transaortic resection approach was chosen in this case due to its ability to effectively address SAM by directly accessing and resecting the anterior mitral leaflet and preferred over others to minimize surgical complexity while adequately managing the LVOT obstruction. The postoperative course and management are discussed. Surgical strategies to prevent LVOTO and SAM are reviewed, highlighting the importance of proper leaflet management and valve orientation to ensure optimal patient outcomes.

## Introduction

Systolic anterior movement (SAM) of the anterior mitral valve leaflet (AML) occurs when the AML moves towards the left ventricular outflow tract (LVOT) and causes left ventricular outflow tract obstruction (LVOTO) [1]. First described in hypertrophic cardiomyopathy patients, this phenomenon has been observed with other structural and non-structural heart conditions. Risk factors for SAM include an aortomitral angle below 120°, anterior leaflet elongation (pathological or postsurgical), an undersized mitral annuloplasty, chordal anomalies or postsurgical chordal repair, anteromedial papillary muscle displacement, bulging septum, and anterior to posterior mitral leaflet height ratio (<1.4) [2].

While several risk factors for SAM exist, in this case, the primary cause was the retained anterior mitral leaflet with intact chordae, leading to late-onset obstruction. LVOTO arises from a complex interaction between left ventricular geometry, leaflet structure, and flow dynamics. AML preservation, as in bileaflet-preserving mitral valve replacement (MVR), can contribute to SAM by altering the normal valve dynamics, leading to LVOTO, which in turn, leads to heart failure by increasing afterload on the left ventricle, which results in impaired left ventricular ejection and a reduced cardiac output. This increased afterload can also cause left ventricular hypertrophy, further exacerbating the obstruction and creating a vicious cycle of worsening heart failure symptoms, as seen in this patient. Managing the AML, often through resection, is crucial to prevent this complication. Though SAM is typically observed in the immediate postoperative period, delayed presentation years after MVR is an uncommon but recognized phenomenon. This case underscores an alternative surgical approach - transaortic AML resection without mitral valve re-replacement - which is less frequently reported but offers a viable solution in select patients, avoiding the risks associated with prosthetic valve re-replacement.

In this report, we highlight a case of SAM secondary to an unresected anterior mitral leaflet occurring several years after mitral valve replacement. A novel approach to resolving the obstruction without re-replacing the mitral valve and managing associated tricuspid regurgitation is described.

## Case presentation

**Figure 1:**
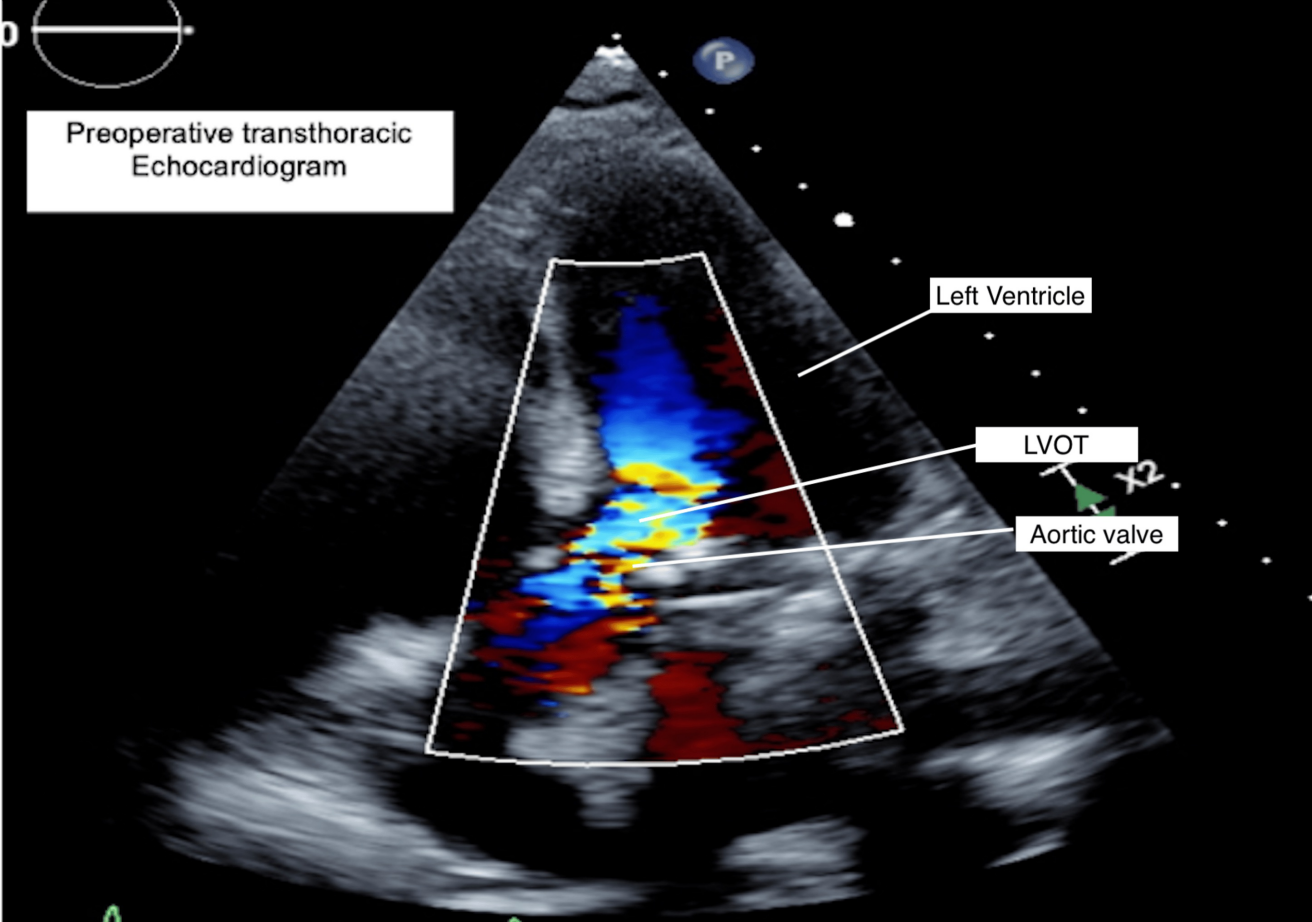
Preoperative Transthoracic echocardiogram showing LVOTO LVOTO: Left ventricular outflow tract obstruction

A 70-year-old female with a past medical history of coronary artery disease, myocardial infarction, chronic heart failure with preserved ejection fraction and atrial fibrillation on coumadin presented with dyspnea, pedal edema, and decreased exercise tolerance suggestive of acute exacerbation of heart failure. Her cardiac history included coronary stenting (six years prior) and mechanical mitral valve replacement six years prior and a dual chamber permanent pacemaker. The mitral valve replacement was reported to have been done with preservation of both mitral leaflets. A transthoracic echocardiogram showed a well-seated bileaflet mechanical mitral valve and dynamic subvalvular LVOT obstruction due to systolic anterior displacement of the anterior mitral leaflet, chordae and papillary muscles with a peak velocity of 4.9 m/s and a mean gradient of 49mmHg (Figure 1). All heart chambers were dilated and there was moderate to severe tricuspid regurgitation. She was referred to the cardiac surgery service following a heart failure exacerbation and a decision was made for surgical repair of LVOT obstruction after medical optimization.

**Figure 2:**
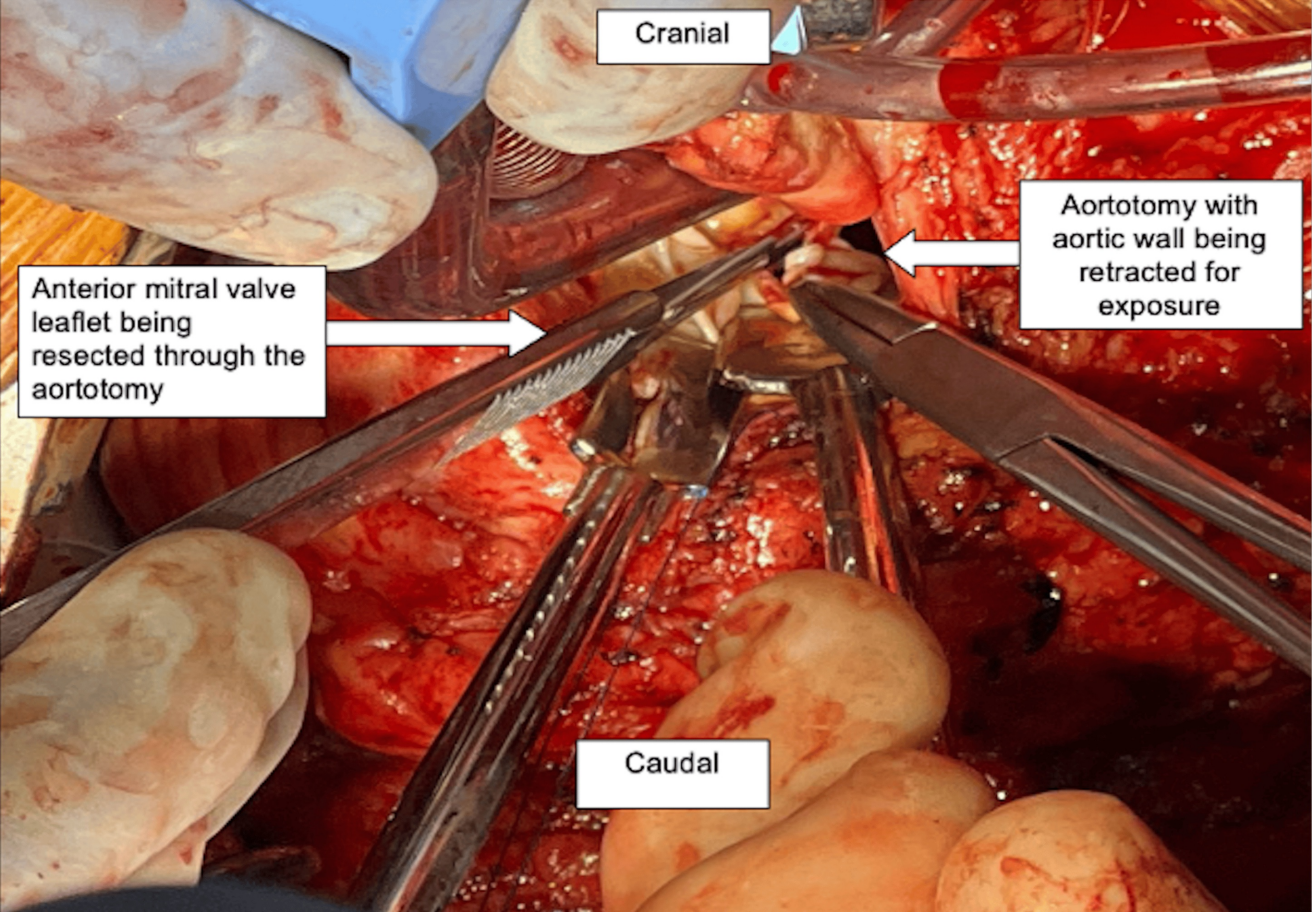
Intraoperative image showing anterior mitral leaflet obstructing LVOTO LVOTO: Left ventricular outflow tract obstruction

**Figure 3:**
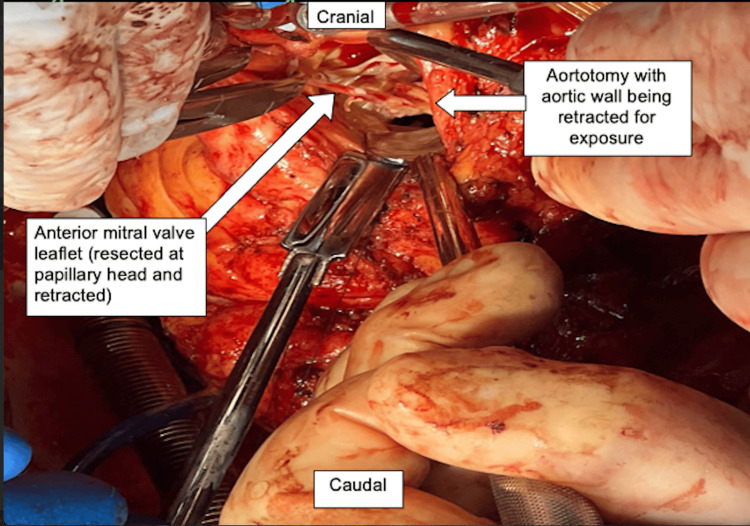
Intraoperative image showing anterior mitral leaflet being resected

**Figure 4:**
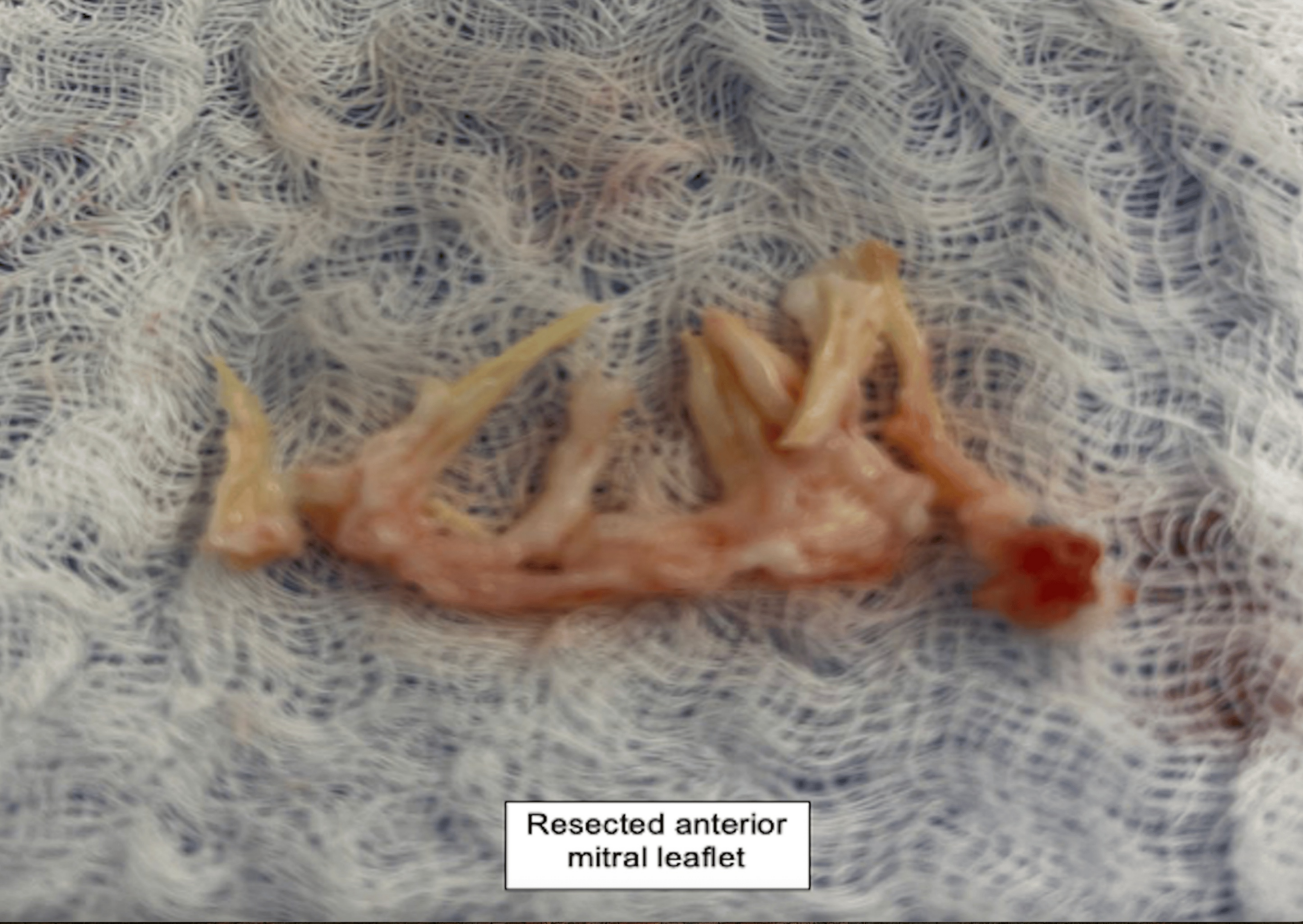
Intraoperative image showing resected anterior mitral leaflet

**Figure 5:**
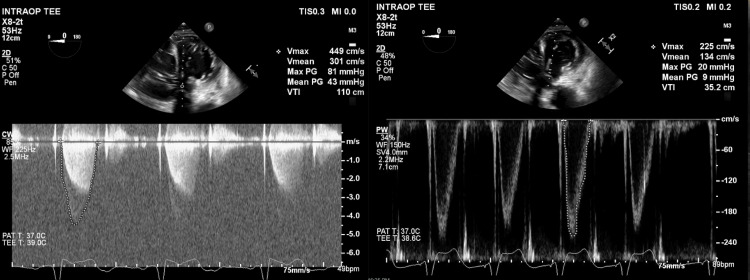
Intraoperative transesophageal echocardiography (TEE) comparing LVOT gradients before (left) and after (right) resection of the AML resulting in improved MG from 43mmHg to 9mmHg Left: Continuous wave doppler across the LVOT before AML resection showing peak and MG of 43mmHg Right: Pulsed wave doppler across the LVOT after AML resection showing reduced MG of 9mmHg LVOT: Left Ventricular Outflow Tract; AML: Anterior mitral leaflet; MG: Mean Gradient

She was taken to the operating room, general anesthesia was induced, and the chest was prepared and draped. She had severe pulmonary hypertension with systolic pulmonary arterial pressures between 70-90mmHg. A redo median sternotomy was performed, systemic heparin was administered, followed by pericardiotomy, adhesiolysis, bicaval and aortic cannulation, initiation of cardiopulmonary bypass and cardioplegic arrest with Del Nido cardioplegia. An oblique aortotomy was made above the sinotubular junction, exposing the mitral apparatus below the native aortic valve (Figure 2). The AML was found to be obstructing the LVOT with intact chordae and papillary muscle attachments (Figure 3). The anterior leaflet was resected (Figure 4) close to the sewing ring of the prosthesis and anterior leaflet chordae were resected to the tip of the papillary muscle, which was spared. The aortotomy was closed and the patient was separated from cardiopulmonary bypass. Intraoperative transesophageal echocardiography showed a decrease in LVOT gradient from 43mmHg to 9mmHg after AML resection (Figure 5). However, moderate-severe tricuspid regurgitation persisted. Cardiopulmonary bypass was reinitiated, a right atriotomy was performed and the tricuspid valve was bicuspidized using a Kay stitch [3] to obliterate the antero-posterior commissure. The atriotomy was closed and the patient was separated from bypass, with mild tricuspid regurgitation. The patient was decannulated, heparin was reversed with protamine, chest tubes were placed, and the chest was closed.

Postoperatively, she was extubated, weaned off inotropes and pressors rapidly, and coumadin was restarted for her mechanical valve. Elevated pulmonary arterial pressures persisted despite inhaled nitric oxide and sildenafil. She continued to do well clinically until her discharge. She remained on sildenafil until discharge.

## Discussion

This case underscores the clinical challenge of post-mitral valve replacement LVOTO and SAM of the anterior mitral leaflet. SAM, which leads to LVOTO, is commonly associated with hypertrophic cardiomyopathy but can also arise from other structural and nonstructural cardiac conditions. LVOTO due to SAM from the retained anterior leaflet has been described [4,5]. This is usually identified early after surgery, and typically has been treated with re-replacement of the mitral prosthesis and resection of the anterior leaflet. 

While this patient may have had the anatomy for SAM and LVOTO postoperatively, it was insidious until her recent acute exacerbation. Severe LVOTO was confirmed, with gradient variability supporting the diagnosis of SAM. Right ventricular function was preserved despite severe pulmonary hypertension. AML resection was preferred over valve re-replacement, posterior leaflet modification, or septal myectomy due to anatomical and surgical considerations. An alternative approach would have been either a left atrial or transseptal approach, especially if a mitral valve replacement was necessary or highly anticipated in addition to the AML resection or plication. Tricuspid regurgitation was linked to annular dilation, and bicuspidization was chosen over ring annuloplasty to minimize prosthetic material and it also has a lower risk of conduction system injury given the location of the plication stitches. Postoperatively, right ventricular function remained stable, no residual LVOT obstruction or SAM was noted, and follow-up included echocardiographic monitoring and pulmonary hypertension management.

Patients undergoing mitral valve replacement tend to have better long-term outcomes when one or both leaflets and their associated chordae and papillary muscles are preserved [6]. However, preservation of these elements adds complexity to the operation, and techniques have evolved to divide and re-orient the anterior leaflet structures [7]. Despite these modifications, Our case is unusual in that the presentation of worsening heart failure symptoms did not occur until several years after the index operation, although the anatomic substrate for SAM must have been present immediately after surgery. It is possible that left ventricular hypertrophy increased over time, exacerbating the LVOTO. The associated tricuspid regurgitation and pulmonary hypertension were likely secondary to prolonged left ventricular pressure overload.

SAM is a rare but recognized complication of leaflet-preserving MVR, typically resulting from dynamic leaflet motion and flow interactions. Doppler, stress echocardiography, and 3D imaging are valuable in diagnosing LVOTO by assessing gradients and leaflet dynamics. While resecting the anterior mitral leaflet via an aortotomy simplifies the procedure, it may not be feasible in cases with pannus formation or subvalvular involvement, and "critical" or "essential" prevention emphasizes the importance of surgical precision to reduce LVOTO risk. Despite relief of LVOTO and tricuspid regurgitation, postoperative pulmonary arterial pressures remained elevated and required aggressive management with pulmonary vasodilators. Ultimately, satisfactory hemodynamics were restored.

This case highlights the importance of recognizing SAM in patients with prior mitral valve replacement and the need for tailored surgical strategies to prevent LVOT obstruction. The preservation of mitral leaflets during valve replacement can predispose patients to SAM and subsequent LVOT obstruction due to altered ventricular dynamics. One strategy to mitigate this risk is dividing the anterior leaflet and anchoring it to its annulus. Excising the central part of the AML from 10-2 o’clock and including the remainder in the valve sutures is a well-described strategy [5]. Careful orientation of the prosthetic valve is also critical. Tissue valves should be oriented to maintain continuity with the aortomitral junction, while bileaflet mechanical valves are often oriented vertically (12- to 6-o'clock position) for sizes ≥27 mm and horizontally (9- to 3-o'clock position) for sizes ≤25 mm [8-10]. This approach aims to optimize blood flow dynamics and reduce the risk of LVOT obstruction. These orientations optimize the spatial relationship between the valve and the LVOT, minimizing the likelihood of SAM.

Intraoperative and immediate postoperative prevention and management of SAM is vital. It entails preload optimization, through careful fluid administration and, if needed, inotropes to reduce obstruction. Beta-blockers should be avoided or used cautiously, as they may lower contractility and worsen the obstruction. Close monitoring of hemodynamic parameters, including LVOT gradients, is essential to guide treatment.

## Conclusions

In conclusion, this case underscores the importance of considering SAM in the differential diagnosis of LVOT obstruction in patients with a history of mitral valve replacement. It emphasizes the need for appropriate surgical techniques and careful postoperative management to ensure optimal patient outcomes. Preservation of leaflet function is advocated during MVR and techniques to manage the AML are established. Failure to adequately divide or translocate the AML can result in LVOTO which can be severe. Echocardiography can define and show resolution of the problem. Surgical repair can be achieved without need to re-replace the prosthetic valve by resecting the obstructing AML through an aortotomy. In this case, transaortic resection of the AML resulted in a decrease in LVOT gradient from 43mmHg to 9mmHg (Figure 5). This may simplify the operation and still achieve adequate relief of LVOTO. Prevention of this complication by following appropriate surgical techniques is mandatory.
